# Role of digital health in coordinating patient care in a hub-and-spoke hierarchy of cancer care facilities: a scoping review

**DOI:** 10.3332/ecancer.2023.1605

**Published:** 2023-09-25

**Authors:** Ramachandran Venkataramanan, Akash Pradhan, Abhishek Kumar, Mohannad Alajlani, Theodorus N Arvanitis

**Affiliations:** 1 Institute of Digital Healthcare, WMG, University of Warwick, CV4 7AL Coventry, UK; 2 Strategy and Research Wing, Karkinos Healthcare, Mumbai 400086, India

**Keywords:** digital health, hub and spoke, telemedicine, neoplasm, cancer, technology

## Abstract

**Background:**

Coordinating cancer care is complicated due to the involvement of multiple service providers which often leads to fragmentation. The evolution of digital health has led to the development of technology-enabled models of healthcare delivery. This scoping review provides a comprehensive summary of the use of digital health in coordinating cancer care via hub-and-spoke models.

**Methods:**

A scoping review of the literature was undertaken using the framework developed by Arksey and O’Malley. Research articles published between 2010 and 2022 were retrieved from four electronic databases (PubMed/MEDLINE, Web of Sciences, Cochrane Reviews and Global Health Library). The preferred reporting items for systematic reviews and meta-analyses extension for the scoping reviews (PRISMA-ScR) checklist were followed to present the findings.

**Result:**

In total, 311 articles were found of which 7 studies that met the inclusion criteria were included. The use of videoconferencing was predominant across all the studies. The number of spokes varied across the studies ranging from 1 to 63. Three studies aimed to evaluate the impact on access to cancer care among patients, two studies were related to capacity building of the health care workers at the spoke sites, one study was based on a peer review of radiotherapy plans, and one study was related to risk assessment and patient navigation. The introduction of digital health led to reduced travel time and waiting period for patients, and standardisation of radiotherapy plans at spokes. Tele-mentoring intervention aimed at capacity-building resulted in higher confidence and increased knowledge among the spoke learners.

**Conclusion:**

There is limited evidence for the role of digital health in the hub-and-spoke design. Although all the studies have highlighted the digital components being used to coordinate care, the bottlenecks, Which were overcome during the implementation of the interventions and the impact on cancer outcomes, need to be rigorously analysed.

## Introduction

Navigating patients through the various levels of a healthcare system and ensuring a continuum of healthcare services are major challenges for most countries [1, 2]. Coordinating cancer care is complicated due to the involvement of multiple service providers and organisations which often leads to fragmentation [3]. It is well known that reforms to coordinate cancer care within health systems can improve the quality of services delivered and the overall patient experience by improving communication between patients and service providers, sharing information with those concerned with the patient, providing referral services, ensuring treatment adherence and reducing the length of stay, waiting time and hospital costs [4].

Towards this end, hub-and-spoke models could be a viable solution to coordinate care [5–7]. The hub-and-spoke model entails centralisation of key resources at a single site where specialised services might be provided and the provision of basic services at secondary sites [7]. Low- and middle-income countries which are experiencing an increasing burden of diseases due to changing demographics and urbanisation are more likely to benefit by adopting the hub-and-spoke design. As inadequate infrastructure is a key concern across these regions, the spokes can leverage the expertise and trained human resources of the resource-rich hubs to provide better services [7]. However, there are multiple challenges that can prevent the creation of an effective network of healthcare facilities. A host of contextual factors including the transportation between hub-and-spoke, congestion at hub and lack of autonomy at spokes might undermine the efficiency even when coordination is achieved [7, 8].

Advancements in technology offer the opportunity to overcome the barriers to efficient working of the hub-and-spoke design. Although the use of the hub-and-spoke model in the healthcare industry is not new, the evolution of digital health implies that care can be provided swiftly at scale even to those residing in remote areas [9]. Global experience indicates that digital health can be used to vastly improve the experience of cancer patients with uses ranging from data collection for research, improved efficiency of diagnostic tests, remote monitoring and teleconsultation of patients and reminders and assistance in decision-making [10–12, 15]. In this regard, digital health is defined as the use of information and communication technologies in medicine and other health professions to manage illnesses, health risks and to promote wellness [16]. Digital health has a broad scope and includes the use of wearable devices, mobile health, telehealth, health information technology and telemedicine [16]. In the context of this study, care coordination is defined as deliberately organising patient care activities and sharing information among all the participants concerned with a patient's care to achieve safer and more effective care.

To our knowledge, there are no studies on the role of digital health in coordinating cancer care in a hub-and-spoke model. Van Hoeve *et al* [4] assessed eight studies to summarise the effect of pathways in oncological care but the discussion on hub-and-spoke design was missing. However, in the same study, the authors found reduced length of stay at the hospital and lower costs for pathway groups. Otty *et al* [17] summarised the literature on optimal care pathway for lung cancer patients but found a limited number of articles on this subject matter. Elrod and Fortenberry [7] have described the Knighton Health System’s service delivery network which has utilised the hub-and-spoke model for over three decades. However, the study is not related to cancer and does not assess the impact of technology. Likewise, the Academic Model Providing Access to Healthcare (AMPATH) oncology model is a comprehensive cancer care model based on a hub-and-spoke design which was initially introduced to control the human immunodeficiency virus (HIV) pandemic. However, the model evolved and today the vast network of spokes is being used for cancer screening activities. The increase in demand for non-acquired immunodeficiency syndrome (AIDS) cancer cases led to planning for infrastructure, transportation, and standardisation of plans and protocols [18].

It is true that the focus on digital health gained more traction during the COVID-19 pandemic. There has been a proliferation of research articles, particularly on the application of telemedicine in the healthcare domain. Despite this growth, there is limited knowledge of the extent to which digital health is being used for providing care for less common diseases such as cancer. Rossman *et al* [20] identified the potential uses of digital health for cervical cancer control in low- and middle-income countries but found the evidence weak with a high risk of bias. Morris *et al* [19] conducted a systematic review of the role of digital health in rural cancer care, but the scope was limited to application in rural areas. Kabukye *et al* [29] conducted a scoping review on the use of digital health in oncology in Africa and found that the long-term impact on cancer-related outcomes needs to be analysed rigorously.

Among digital health, telemedicine is the most popular medium to diagnose, treat and support cancer patients, especially to ensure access to those residing in remote areas [19, 20]. There are a couple of studies that provide a narrative review of the possible use of telemedicine in cancer care [11, 21]. More recently, a review of the role of telemedicine in providing palliative care found that telemedicine can provide timely service to patients and reduce the need for in-patient visits which is difficult due to reliance on caregivers and restricted mobility. A narrative review by Gibelli *et al* [22] reported that telemedicine can lead to improved patient management and provide faster and more information to patients suffering from thyroid cancer. Zhang *et al* [12] also highlighted the use of mobile technologies to create awareness and promote screening for cervical screening.

Notwithstanding the limited evidence, healthcare systems across the world are adopting digital health. Although there are studies evaluating the working of hub-and-spoke models and the impact of digital health, these are two different strands of literature. Certainly, there are no reviews on the technology-enabled hub-and-spoke models for coordinating cancer care. Realising this gap, the study aims to synthesise the existing evidence on the uses of digital health in coordinating cancer care within the hub-and-spoke framework. The key barriers and facilitators to the use of digital health are discussed and the impact on outcomes is highlighted.

## Data and methods

Our scoping review followed the five-stage framework developed by Arksey and O’Malley [13]: 1) identifying the research question(s); 2) identifying relevant studies; 3) selecting the studies for review; 4) charting the data from them and 5) collating, summarising and reporting results. The PRISMA-ScR checklist was followed to present the findings [14].

### Identifying the research question

This scoping review aims to study the role that digital health can play in coordinating cancer care in a hub-and-spoke model. More specifically, the following research questions were investigated:

1. What are the different ways in which digital health is being used in hub-and-spoke models to coordinate the delivery of cancer care services?
2. What are the facilitators and barriers to the use of digital health within the hub-and-spoke models?
3. What is the impact on patient and health system outcomes?

### Information sources and search strategy

Electronic databases through which the search was carried were PubMed/MEDLINE, Cochrane Library, Web of Sciences and Global Health Library. In addition, Google searches using Google Scholar were conducted. A preliminary search was conducted using PubMed and Web of Sciences based on which the key terms for the search were identified after screening the abstract titles and relevant research articles. The keywords and mesh terms identified using PubMed are presented in Table 1. A combination of these keywords was used for conducting the search using the various databases. The references of the shortlisted articles were backtracked to identify additional research papers and relevant literature.

### Study selection

Following the search, all the data were collated using MS Excel and duplicates were removed. Two reviewers went through the abstracts of the articles and identified the relevant articles based on the inclusion and exclusion criteria. Only those articles which met the criteria were retrieved in full and were further evaluated for their relevance based on text. Reasons for exclusion at each stage were recorded in Excel itself. The PRISMA-ScR checklist was followed. The inclusion and exclusion criteria are as follows.

### Inclusion criteria

**Participants.** The review included studies involving patients who are suffering from cancer and healthcare providers involved in organising healthcare activities for cancer patients.

**Concept.** Any application of digital health in cancer care within the hub-and-spoke design framework was considered. Here, digital health refers to the use of information and communication technologies to manage and provide cancer care and to promote wellness. During preliminary research, most of the articles that were identified were related to telemedicine which is defined as the use of telecommunication technology to diagnose and treat patients.

**Context.** The studies included in this review focus on the use of digital health to support the diagnosis and treatment of patients and ensure the well-being of patients by organising patient care activities.

### Type of sources

The scoping review considered both quantitative as well as qualitative studies. Only articles published in English were considered. A preliminary search using MEDLINE showed that there were very few articles on this subject matter prior to 2010. Therefore, articles published after 2010 were included. Reviews and editorials on related topics were also included.

### Exclusion criteria

Studies not related to the application of digital technology for coordinating cancer care were excluded. Studies that do not involve reference to the hub-and-spoke design were excluded, and, finally, non-peer-reviewed articles were excluded.

### Data charting process

Data were presented with an aim to provide a descriptive summary that is aligned with the objectives of this review. Two independent reviewers were involved in the data-charting process. A custom-made template in Excel was used. The data chart included the details about the study such as the year, authors, title, region and so on. Specific details about the use of technologies and the type of cancer for which care is being sought, the number of spokes and hubs included in the study design are also provided. In case any information was missing, the authors of the articles were contacted to fill in the gaps.

### Synthesis of results

Using the narrative approach, the selected studies were reviewed, and results were reported in terms of key aspects of the digital health interventions. The extracted data were presented in tabular form (Table 2). A total of seven studies were shortlisted.

## Results

**Figure 1.**
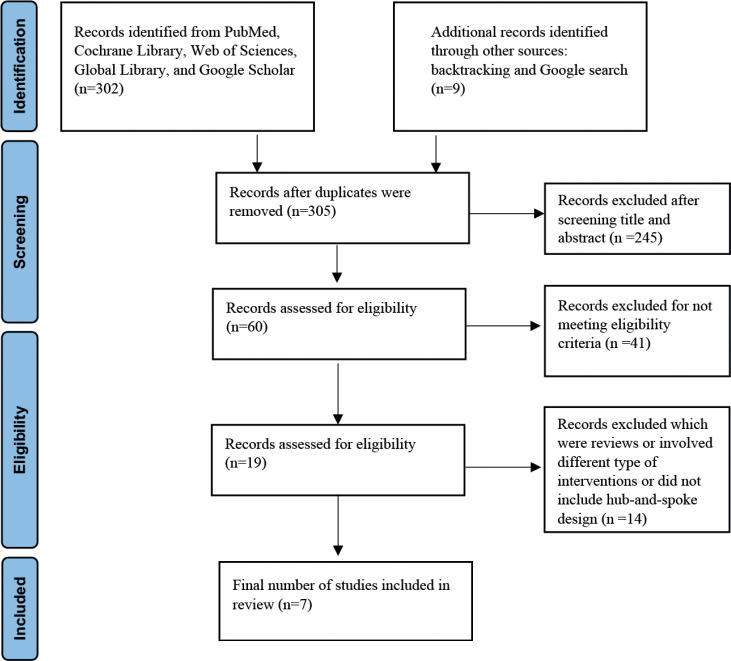
PRISMA flow diagram.

The search of published literature in databases resulted in 302 articles (Figure 1). In addition, nine articles were retrieved through other sources. After removing duplicates, 305 remained, of which 245 were excluded after screening the title and abstract. Of the 60 remaining articles, only 19 met the inclusion criteria. Further, 14 studies were excluded which though related to cancer and were aimed at coordinating cancer care through digital health did not include hub-and-spoke design. A total of seven studies were included for the final review which indicates that there are very few studies that aim to understand the impact of using digital health on cancer care coordination within a hub-and-spoke framework.

### Articles characteristics

Of the seven studies, one was conducted in 2013 [24] and two in 2017 [25, 37] while the rest four were conducted during 2021–22 [23, 26–28].

Two of the studies identified were related to the Extension for Community Healthcare Outcome (ECHO) model which involved connecting the health workers at spoke sites with experts at the hub using videoconferencing [27, 28]. These studies aimed to assess the educational outcomes of the programme and to gain a better understanding of the reasons individuals choose or decline to participate in the Cancer ECHO programme. The programme involved weekly and biweekly sessions, which enabled the participants at the spoke site to discuss specific cases with the experts. The sessions also included didactic presentations by experts on cancer care. Two of the studies in the US and Canada aimed to address how the care gap for cervical and thoracic cancer in rural areas could be bridged through telemedicine [24, 25]. One study in the US explored the feasibility of providing occupational therapy to breast cancer patients through telemedicine [26]. Another study in the US conducted by Harris *et al* [37] investigated whether a technology-enabled hub-and-spoke model could support peer review in radiation oncology and lead to standardisation of cancer care across facilities. The study based in India described the implementation of a hub-and-spoke model for cancer care and the use of digital applications for risk assessment and navigating patients across facilities [23].

Two studies followed a mixed methods approach [24, 25], four were observational studies [23, 27, 28] and one was a prospective study [26]. Overall, the studies highlighted that technology can be used for capacity building, providing specialised care in remote areas, and to navigate patients to facilities for diagnosis and treatment.

### Evaluation of studies

This section provides details about the digital interventions which were used to improve access to specialised cancer care services and to coordinate care (Table 2). Hitt *et al* [24] examined the working of a telemedicine programme that was rolled out in Arkansas to deliver tele-colposcopy services to patients residing in rural areas who were being screened for cervical cancer but did not have access to specialists who could diagnose based on results of the screening examination. The intervention included delivery of colposcopy services via interactive telemedicine at four separate spoke sites where a trained nurse collected specimens under the supervision of an expert on a real-time basis. The intervention led to an improvement in the number of meetings scheduled for a colposcopy examination.

Hitt *et al* [24] reported that between 2010 and 2011, 1,812 visits were scheduled for patients across 68 counties at the four spoke sites and 1,298 tele-colposcopy exams were conducted. The respondents reported that the intervention drastically cut the waiting time. In the absence of the intervention, most of the patients (60%) would have waited for 6–12 months to seek care. Data on the characteristics of the patients revealed that approximately 40% of the patients were smokers and the average age at first intercourse was 16 years. The novelty of the intervention lies in the fact that the nurses were recruited locally before the intervention and were provided training for performing tele-colposcopy. However, the analysis in the study is based on data for 2010–2011 only which calls for rigorously analysing the data over a longer period.

Realising that the spokes only had one radiotherapist, Harris *et al* [37] presented a peer review model in which five community centres in rural areas (spokes) partnered with Leo Jenkins Centre at East Carolina University with an aim to review radiotherapy plans and recommendations made at the spoke. The model involved online meetings which were conducted thrice a week between 8 and 9 am using Webex, video- and phone-based programmes. All the partners from the hub and spoke participated with an aim to achieve standardisation of care across the facilities. Comparison of data for 283 cases at baseline and 352 case after 1 year revealed a 50% decline in prospective radiotherapy treatment plans which required modifications.

A similar study from British Columbia, Canada evaluated the role of telemedicine in providing thoracic oncology care to patients residing in remote areas [25]. The study reported that prior to the intervention the experts used to travel to the site from the tertiary facilities to see patients. Patients would wait for up to 6 months for the experts at the facilities. The intervention which was subsequently rolled out involved telemedicine consultation through virtual clinics. The components of the intervention involved a videoconferencing network, high-resolution remotely-controlled Zoom camera, a document reader (analogue to digital) and the availability (at some sites) of an electronic stethoscope. Between 2003 and 2015, 3,897 telemedicine community visits took place and the number of telemedicine encounters increased from 320 to 1,709 which resulted in saving a lot of time for patients residing in rural areas. An average distance of 766 km was saved per patient. The study does not provide estimates of the implementation cost which was mostly borne by local health authorities.

Another study assessed an intervention which was rolled out in the US and was targeted at providing access to breast surgery occupational therapy through telemedicine [26]. The intervention involved the use of videoconferencing to connect patients at spoke sites with therapists at the hub. The patients attended the session at the spoke site which is 75 km away from the hub using a computer system enabled for video conferencing. Also, there was an assistant at the spoke site who worked under the supervision of the therapist, but information is not provided on whether the assistant received any training prior to the intervention. The intervention helped the patients reach baseline function within an average of 42 days of surgery for breast cancer. However, of the 26 patients enrolled only 18 completed postoperative sessions in which functional assessments, exercises and education were provided. In this study, sample size is very small and the reason for the attrition of the patients is not mentioned.

Two studies were based on the ECHO model, and both were published in 2022 [27, 28]. The programme is aimed at capacity-building and sharing best cancer care practices with health care workers at the spoke sites. In this model, the participants at hub and spoke sites are connected through videoconferencing. The participants meet on a weekly or bi-weekly basis. While the experts at the hub are involved in capacity-building, the spoke participants discuss de-identified cases to further their understanding about care aspects. Milgrom *et al* [27, 28], through these studies, have attempted to identify the reason for the participation of the healthcare professional as well as their knowledge outcomes. The studies are based on 22 interviews and 30 one-time surveys. Results indicate that the programme could be instrumental in building capacity, particularly in rural areas where there is a shortage of specialists. Milgrom *et al* [27] found that the reason for non-participation in the programme was the lack of time and length of the sessions. However, in another study, the same authors found that participants were satisfied with the programme and appreciated the conversation format, support and experience gained through the programme [28].

Ramachandran *et al* [23] described the working of a distributed cancer care model which was conceptualised with an aim to generate the demand for cancer screening and to overcome the barriers related to access to healthcare facilities in Kerala, India. The key characteristic of this model was the use of technology to serve patients residing in rural areas and to guide junior oncologists at spoke centres. In this model, initially screening camps were organised across two districts in Kerala where prospective patients at risk of developing cancer were assessed for the risk of developing cancer using a mobile phone-based application. Furthermore, the patients at risk were navigated to spokes for consultation and diagnosis by a centralised system (command centre). Further patients who require chemotherapy and surgery were navigated to partner hospitals (hub and spokes) using digital applications. The entire process of patient navigation was orchestrated by the centralised system which also keeps a digital record of patients. However, the analysis in this study is based on data for five months (July to December 2021) and the implementation is still in the pilot phase. Moreover, the study does not include a control group with which the outcomes could be compared. A further rigorous evaluation of the model is required to understand the impact on early detection of cancer and mortality outcomes.

## Discussion

The scoping review was conducted with an aim to summarise the use of digital health in coordinating cancer care via hub-and-spoke models. The review aims to highlight the key digital health components used to achieve coordination between facilities and the impact on outcomes such as access and treatment success. Due to the limited number of studies on this subject matter which were based on the experience of the US, Canada and India, the review could not identify the context for rolling out the intervention. The review, however, identifies the key components of digital health which helped to mitigate the problem of access to diagnosis and specialised care.

In line with some of the existing reviews on digital health technology for cancer care, telemedicine through videoconferencing was identified as the main technology which is being used to coordinate the delivery of care and to conduct capacity-building sessions [15, 19]. The use of telemedicine in the selected studies was synchronous (live exchange of patient information) as well as asynchronous (store and forward patient information). It should be noted that apart from videoconferencing there are several other components of digital health such as SMS and phone calls which could be used to book appointments, send reminders to patients, create awareness by posting content,and providing counselling and decision support for self-monitoring [19, 29]. In addition, electronic health records are widely used to track the journey of cancer patients across facilities and to reduce the waiting time for diagnosis and treatment [30, 31]. Recently, some studies have reported that mobile phone-based imaging devices can be used to screen individuals and shared via cloud platform for remote specialist review [33, 34]. However, none of the identified studies in this review were based on these applications of digital health.

In our review, the studies focused on breast, thoracic and cervical cancer. The geographical coverage in the selected studies was quite narrow with the maximum number of spokes being 63 in one of the studies [25]. Further research is required to understand the reason for low-scale adoption since considerable positive externalities can be generated by increasing the geographical coverage of the interventions where the infrastructure and resources are inadequate. Further, it was observed that the major barrier to digital-based interventions could be the cost of setting up the infrastructure. Although Hitt *et al* [24] estimate the cost to be around $40 per exam which involved one physician and four nurses and assistants, further research on costing aspects is required as it may vary depending upon the settings and context.

Overall, the benefits from the interventions in the selected studies were observed in terms of time saved, access to specialised care, navigation of patients across facilities, standardisation of cancer care plans, improvement in outcomes and increase in the use of telemedicine at the spokes [23–26, 37]. Some of the previous studies have also highlighted that increase in coordination across facilities could result in less travel for patients as well as doctors, improved access to treatment and preparation of better treatment plans which also led to reduction in waiting time and timely delivery of treatment and patient being treated in their own community [4, 6, 15]. However, for certain specialised services such as chemotherapy and surgery patients might be required to travel to the hub. In the AMPATH model, transportation services were arranged between spokes and hub so that the barrier to access could be overcome [18]. Clearly, setting up the infrastructure which could cater to the increasing demand is a key requirement for the optimal working of the hub-and-spoke model.

One important finding that emerges from the review is the increase in encounters over time [25]. Certainly, it can be presumed that as both patients and healthcare providers become increasingly accustomed to and proficient with technology, the quality of communication using digital mediums is likely to improve progressively. Countries where the subscription of internet and mobile services is increasing can expect similar results once the interventions are rolled out. It is believed that better patient management using telemedicine could lead to an improvement in survival rates by 5%–10% [21]. However, none of the studies identified in this scoping review evaluated the survival outcomes.

The facilitators across the selected studies ranged from training the nurse prior to the intervention, funds being allocated to set up the network at the hub and spoke sites by the local health authorities; setting up of a cloud-based IT system and the availability of trained assistants at the spoke sites in the remote occupational therapy services [24–26, 37]. While patient satisfaction was generally high, the studies did not address potential barriers, such as patient technology usage.

Towards this end, findings from a recent study that evaluated the Queensland remote chemotherapy model aimed at providing chemotherapy through videoconferencing found that the major barriers to effective implementation of the model were frequent turnover of senior management and nursing staff at the facilities and internet disruption during videoconferencing [35]. Findings from the evaluation of the Queensland remote chemotherapy model and Townsville Tele-oncology Network indicate that to ensure smooth delivery of chemotherapy services in rural areas it is recommended that common guidelines be developed and adequate resources be provided to rural areas [35, 36].

In our review, the telemedicine services were mostly used by health workers at spokes and hub. In remote areas, limited skills among healthcare providers and patients may result in underutilization of technology. Further research need to analyse the geographic variation in use of digital health [32].

## Limitations

The results of the scoping review might be interpreted with a little caution because the sample size of the studies as well as the period over which the data were analysed was small. Furthermore, the difference in methodology and the nature of cancer across the studies varied which calls for analysing the outcome over a longer period for site-specific cancers. Another issue is the absence of cost estimates in the studies. Future research should include cost-effectiveness data, aiding countries with budget constraints in their planning and evaluation. Additional details about the number of specialists and health workers were not available which prevents us from understanding the true burden on the health care system particularly at the spokes and the context for rolling out the interventions. The increase in demand for services could lead to crowding at the spoke sites which can affect the delivery of services and needs to be examined further.

## Conclusion

This scoping review was conducted to synthesise knowledge on the working of hub-and-spoke models which are using digital health to bridge the cancer care gap for patients. Despite the limited evidence which mostly pertain to the US, Canada and India, we were able to present a working of the existing digital health which would be helpful for rolling out similar initiatives in countries where patients do not have access to specialised care. Although all the studies have highlighted the pathways, the bottlenecks, which were overcome during the implementation and the impact on cancer outcomes, remain unclear and need to be rigorously analysed in the future. Future studies are required to investigate the impact of introducing various digital health interventions, particularly in low- and middle-income countries which are slowly embracing technological changes in the healthcare domain.

## Conflicts of interest

No potential conflict of interest was reported by the authors.

## Funding

This research was conducted without any external funding support.

## Author contributions

Ramachandran Venkataramanan managed the production of this paper, leading the drafting and data analysis processes. Akash Pradhan and Abhishek Kumar contributed to the scoping process, drafting the manuscript and data analysis. Mohannad Alajlani designed the scoping review and contributed to defining the objectives, parameters and methodology used within the study. Theodorus N Arvanitis supervised the project provided the direction for the project and was central to developing the scoping review paper.

## Figures and Tables

**Table 1.** Search terms used for preliminary database searches.

Cancer care		Hub-and-spoke model		Digital health
‘neoplasms’ [MeSH Terms]	AND	‘hub-and-spoke’	AND	‘Telemedicine’ [MeSH Terms]
‘cancer care’		‘hub’ and ‘spoke’		‘mhealth’
‘integrated cancer care’		‘hub and spoke’		‘Mobile’
‘cancer patient management’				‘Digit Health’
‘cancer patient navigation’				
‘cancer care pathway’				

**Table 2.** Description of study population and components of the intervention.

S. No	Author, year and country	Title	Study design	Number of hubs and spoke	Intervention description	Cancer type, participants and sample size	Digital technology components used	Outcomes
1	Hitt et al, 2013 [24], Arkansas, United States	Telemedical Cervical Cancer Screening to Bridge Medicaid Service Care Gap for Rural Women	Observational study	One hub (Little Rock) and four spoke sites (county health units at Hempstead County, Johnson County, Cross County and Desha County)	During each weekly 3-hour clinic, an advanced practice nurse/nurse practitioner at each of the four spoke sites, performs colposcopy and collects biopsy specimens under the real-time interactive supervision of obstetrics-gynaecology faculty member at the hub site in Little Rock	Cervical cancer. Participants are women residing in rural areas who received Pap smears through local County Health Unit. 1,812 telemedicine encounters were conducted between January 2010 and June 2011 which covered patients across 68 counties.	Colposcopy services via interactive telemedicine	• 1,118 sets of biopsy specimens were collected of which 29.8% (333) showed precancerous lesions or cancer, 382 showed mild dysplasia and 403 were benign.
2	Harris *et al*, 2017 [37], United States	A prospective peer review model for radiation therapy	Observational study	One hub (Leo Jenkins Cancer Center at East Carolina University, Greenville, NC, USA) and five spokes (community clinics: The Outer Banks Hospital; Roanoke Chowan (Ahoskie) Radiation Therapy; Beaufort Radiation Therapy Onslow Radiation Oncology and Vidant Radiation Oncology (Greenville)).	The radiotherapy treatment plan and recommendations are reviewed by peers at the hub and spokes during a meeting where participants from hub and spokes are invited. The online meetings are conducted thrice a week between 8 and 9 am using Webex, video and phone-based programmes.	283 cases at baseline and 352 cases after one year. All the eligible cases from the spokes were considered during the review process which required radiotherapy. Participants in the meeting were radiologists and dosimetrists from hub and spokes.	Webex, video and phone-based programme.	• Due to the implementation of the peer review process, there was a 50% decline in prospective radiotherapy treatment plans which required modifications.
3	Humer and Campling, 2017 [25], British Columbia, Canada	The role of telemedicine in providing thoracic oncology care to remote areas of British Columbia.	Observational study	One hub (Kelowna) and 63 spoke sites	Videoconferencing network allows experts at the hub site to access patient records and radiology images at the spoke site.	Thoracic cancer. Patients referred for any thoracic condition.	Telemedicine services and PACS through a secure videoconferencing network. Technical equipments included remotely controlled Zoom camera, document reader and electronic stethoscope	• 15,073 telemedicine encounters were conducted between 2003 and June 2015 of which 50% were follow ups. • On average, 766 km was saved per patient.
4	Lai et al, 2021 [26], United States	Feasibility of Remote Occupational Therapy Services via Telemedicine in a Breast Cancer Recovery Programme.	Prospective Study	One hub (main medical centre) and one spoke site (community practice cancer centre) which are 75 km apart	With help of videoconferencing the therapist at the hub conducts the session for the participants at the spoke site. The therapy session takes place under the supervision of a rehabilitation aide at the spoke site who also performs the physical assessment of the patients.	Breast cancer patients (N = 26).	Telemedicine via videoconferencing	• Telemedicine encounters increased from 320 to 1,709 between 2004 and 2015. • Patient travelled on average 16 miles to reach the spoke. • The average number of telemedicine sessions attended per patient was 3.0 session. • The average time to complete recovery was 42.4 days after surgery
5	Milgrom *et al*, 2022 [27], United States	An evaluation of an ECHOs intervention in cancer prevention and survivorship care	Mixed methods study	Not mentioned	ECHO is a tele-mentoring programme in which the experts at the hub, with an aim to augment capacity, lead discussions on cancer-related topics and cases presented by spoke participants. 22 sessions were conducted which were typically 1.5 hours in length and consisted of a 20-minute didactic presentation and a 1-hour case discussion.	All types cancer. For structure interviews: 15 programme participants (12 spokes learners and 3 hub members) and 7 potential spoke participants who were aware of the programme but did not participate (*N* = 22). In addition, 30 one-time surveys were conducted.	Tele-mentoring programme via videoconferencing	• On the five-point scale, the spokes learners gave higher ratings to their improvement in knowledge, confidence and practice.
6	Milgrom et al, 2022 [28], United States	Enhancing cancer prevention and survivorship care with a videoconferencing model for continuing education: a mixed-methods study to identify barriers and incentives to participation	Mixed methods study	Not mentioned	ECHO is a tele-mentoring programme in which the experts at hub, with an aim to augment capacity, lead discussions on cancer related topics and cases presented by spoke participants. 22 sessions were conducted which were typically 1.5 hours in length and consisted of a 20-minutes didactic presentation and a 1-hour case discussion.	All types cancer. For structure interviews: 15 programme participants (12 spokes learners and 3 hub members) and 7 potential spoke participants who were aware of the programme but did not participate (N = 22). In addition, 30 one-time surveys were conducted.	Telelearning continuing education programme via videoconferencing	• Incentives identified included the programme’s high-quality design, supportive learning climate and access to information. • Barriers included a lack of external incentives to participate and limited time available.
7	Ramachandran *et al*, 2022 [23], India	A Distributed Cancer Care Model with a Technology-Driven Hub-and-Spoke and further Spoke Hierarchy: Findings from a Pilot Implementation Programme in Kerala, India	Observational study	One hub (Rajagiri Hospital, Ernakulam) and four spoke sites (Chazhikattu Multi-Specialty Hospital, Idukki, Mar Besilios Medical Mission Hospital, Ernakulam, Munnar Tata hospital, Idukki and SD Tata hospital, Ernakulam)	Screening camps were organised at a district level to identify at-risk population using digital applications. Patient navigated to spokes and hub for further guidance, diagnosis, and treatment by a centralised system (command centre) using mobile and digital applications.	Four types of cancer- breast, cervix, oral and colon. A total of 2,459 screened for cancer at spokes.	Mobile and web-based applications for risk assessment and patient navigation	• Of 2,499 patients, 12% (299) were screened positive and 189 received chemotherapy session.
